# Inhibition of TTR Aggregation-Induced Cell Death – A New Role for Serum Amyloid P Component

**DOI:** 10.1371/journal.pone.0055766

**Published:** 2013-02-04

**Authors:** Karin Andersson, Malgorzata Pokrzywa, Ingrid Dacklin, Erik Lundgren

**Affiliations:** 1 Department of Molecular Biology, Umeå University, Umeå, Sweden; 2 Institute of Biomedicine, University of Gothenburg, Gothenburg, Sweden; “Mario Negri” Institute for Pharmacological Research, Italy

## Abstract

**Background:**

Serum amyloid P component (SAP) is a glycoprotein that is universally found associated with different types of amyloid deposits. It has been suggested that it stabilizes amyloid fibrils and therefore protects them from proteolytic degradation.

**Methodology/Principal Findings:**

In this paper, we show that SAP binds not only to mature amyloid fibrils but also to early aggregates of amyloidogenic mutants of the plasma protein transthyretin (TTR). It does not inhibit fibril formation of TTR mutants, which spontaneously form amyloid *in vitro* at physiological pH. We found that SAP prevents cell death induced by mutant TTR, while several other molecules that are also known to decorate amyloid fibrils do not have such effect. Using a *Drosophila* model for TTR-associated amyloidosis, we found a new role for SAP as a protective factor in inhibition of TTR-induced toxicity. Overexpression of mutated TTR leads to a neurological phenotype with changes in wing posture. SAP-transgenic flies were crossed with mutated TTR-expressing flies and the results clearly confirmed a protective effect of SAP on TTR-induced phenotype, with an almost complete reduction in abnormal wing posture. Furthermore, we found *in vivo* that binding of SAP to mutated TTR counteracts the otherwise detrimental effects of aggregation of amyloidogenic TTR on retinal structure.

**Conclusions/Significance:**

Together, these two approaches firmly establish the protective effect of SAP on TTR-induced cell death and degenerative phenotypes, and suggest a novel role for SAP through which the toxicity of early amyloidogenic aggregates is attenuated.

## Introduction

Serum amyloid P component (SAP) is a plasma glycoprotein. It is a member of the pentraxin superfamily of calcium-dependent ligand binding lectin proteins. Another member is C-reactive protein (CRP), the classical acute-phase reactant in humans [1], [2]. These proteins have been highly conserved throughout vertebrate evolution. There is a considerable degree of sequence homology (51% identity, 66% homology) within the pentraxin family, although the proteins are functionally distinct [3], [4]. The biological role of SAP is only partly known. It binds in a calcium-dependent way to molecular arrays such as DNA, chromatin, histones, and phosphoethanolamine-containing membranes, suggesting that it has a function in clearance of late apoptotic cells [5]–[10].

Amyloidosis designates diseases involving the deposition of fibrillar proteins, mainly in extracellular spaces. Accumulation of amyloid fibrils is associated with cellular dysfunction and cell death [11], [12]. At least 27 different human proteins are known to cause amyloidosis [13], the best known being the Aβ peptide (which is associated with Alzheimer’s disease), prion protein, and transthyretin (TTR).

TTR is found in blood plasma, the cerebrospinal fluid, and the vitreous humor of the eye. It is composed of four identical subunits of 127 amino acids rich in β-structures [14]. Its biological role in the transport of the thyroid hormone thyroxine and of retinol (via retinol-binding protein) is well established [15], [16].

TTR is deposited as amyloid fibrils in three pathological conditions. Senile systemic amyloidosis occurs mainly in males at advanced age, with predominant manifestation in the heart due to deposits of wild-type TTR [17]. Familial forms of TTR-related amyloidosis are inherited in an autosomal dominant manner and arise from single point mutations in the coding sequence of the TTR gene. These forms of TTR-related amyloidosis are characterized by deposition in several organs including the intestine, the vitreous body of the eye, and particularly along peripheral nerves. The typical manifestation is polyneuropathy, which affects motor, sensory, and autonomous functions; this form has therefore been termed familial amyloid polyneuropathy. However, some mutations in the TTR gene are associated with amyloid deposits found mainly in cardiac tissue, which lead to familial amyloid cardiomyopathy (for review, see [18]).

Amyloid consists of aggregated proteins with an ordered fibril structure. In addition, all types of amyloid are decorated with a number of non-fibrillar constituents, the identity of which is essentially independent of the precursor protein making up the fibrils. These universal non-fibrillar components include SAP [19] and glycosaminoglycans, especially heparan sulfate, dermatan sulfate, and chondroitin sulfate [20]. The amount of SAP in amyloid deposits might be remarkably high, as up to 20 g of SAP per kg dry weight of amyloid fibril has been reported (even though the plasma pool of SAP remained unchanged) [21]. The exact role of these molecules in amyloidogenesis is unknown; none of the structural details of their interaction with amyloid fibrils are known either. However, their universal presence in all known deposits suggests that they have a functional role with as yet unknown clinical consequences.

Reports on the role of SAP in amyloid formation are contradictory, since both inhibition [22] and promotion of aggregation [23], [24] have been shown in the case of the Aβ peptide of Alzheimer’s disease. Since SAP is the major acute-phase reactant in mice, lipopolysaccharide (LPS) can be used to trigger its production. However, forced expression of SAP by LPS has not been found to increase the formation of TTR amyloid in transgenic mice carrying the human TTR gene [25]. Interestingly, mice deficient in SAP develop Aβ amyloid that is similar histochemically to deposits in normal mice, although amyloid formation is delayed and its quantity reduced [26].

More than 100 mutations of TTR are known to date (http://www.bumc.bu.edu/Dept/Content.aspx?DepartmentID=354&PageID=5530). Most of them are associated with polyneuropathy. It is established that the mutations in the TTR gene destabilize the native homotetramer [27], [28], which is accompanied by formation of toxic oligomers and later mature amyloid [29]. Previous studies have shown that similarly to the wild-type TTR, clinical mutants (e.g. TTRV30M) form stable tetramers *in vitro* unless incubated under mildly acidic conditions. We have constructed two TTR mutants, one in the edge region comprising the short β-strand D, denoted TTR-D (G53S/E54D/L55S) [30] and the other in the neighboring β-strand A, denoted TTR-A (V14N/V16E) [31]. These mutants are excellent tools for studies of amyloid-induced cellular toxicity since they spontaneously form protofibrils in a reasonable time period at physiological pH.

In this work, we have shown that SAP has a protective effect in cell culture during early aggregate formation, which protects from TTR-induced cell death. To determine the role of SAP in TTR-induced toxicity, we complemented the *in vitro* studies with a genetic approach in a *Drosophila* model for TTR-associated amyloidosis [32], [33]. In the fruit fly, overexpression of the mutated variant TTR-A in secreted form leads to a complex neurological phenotype that reflects several features of the human pathology, including progressive neurodegeneration, accumulation of insoluble TTR, locomotor dysfunction, and premature death. We have found an increased aggregation rate and toxicity of TTR-A in the fruit fly, which results in an abnormal wing posture termed “dragged wings”. This phenotype is significantly suppressed in crosses with transgenic SAP flies. In addition, we have found *in vivo* that binding of SAP to mutated TTR-A in the eye of *Drosophila* protects retinal structure from the deleterious effects of aggregating amyloidogenic TTR.

## Results

### Binding of SAP to Pre-fibrillar Aggregates of TTR

**Figure 1:**
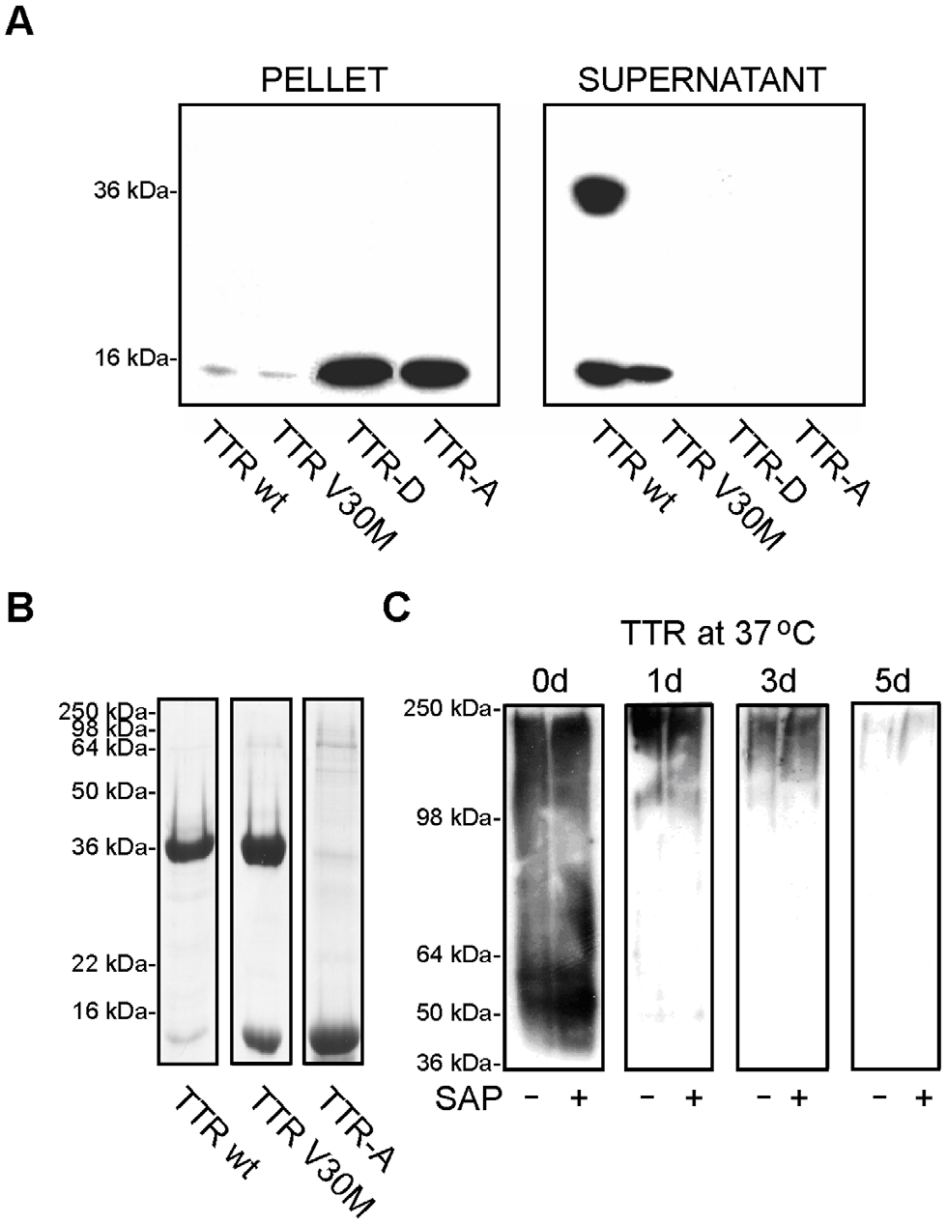
SAP binds to pre-fibrillar aggregates of TTR *in vitro*. *(A)* SAP was co-incubated with pre-aggregated TTR under physiological conditions. The complexes were immunoprecipitated with a SAP-specific antibody (DAKO) and the presence of TTR was detected on immunoblots using a polyclonal anti-TTR antibody (DAKO). SAP bound to pre-fibrillar aggregates of TTR-D and TTR-A, and the precipitates were found in the pellet fraction (left panel), whereas TTR wt and TTR V30M were found unbound in the supernatants (right panel). Bands: 16 kDa–monomer; 36 kDa–dimer. *(B)* SDS-PAGE analysis of TTR variants. Immunoblot shows that the TTR-A mutant is sensitive to SDS and easily dissociates into monomers in contrast to TTRwt or TTRV30M that keep the dimers intact. (*C*) Effect of SAP on aggregation of TTR. The TTR-A mutant was aggregated at 37°C for 0–5 days in the presence (+) or absence (−) of 3 µM SAP and subjected to immunoblotting under native conditions. TTR was detected with a TTR-specific antibody. SAP did not affect the aggregation kinetics of the TTR-A mutant, since the migration pattern of TTR-A in the gel decreased with time as the protein formed higher-molecular-weight aggregates–and was identical irrespective of whether or not SAP was present. After 5 days, the TTR-A formed aggregates that did not enter the separation gel.

It is well established that SAP is commonly found in different types of amyloid deposits and that it has also a calcium-dependent affinity for binding to isolated mature amyloid fibrils. In previous work, we showed that the toxic effect found in cell culture correlates best with the early stages of fibril formation and that the mature full-length TTR fibrils represent an inert end stage [34]. In order to investigate whether binding of SAP occurs early, before the fibrils are formed, we subjected recombinant TTRs to aggregation at physiological pH in the presence of SAP for 4 days at 37°C. Under these conditions, TTR-D and TTR-A mutants are known to form pre-fibrillar aggregates *in vitro*, whereas TTRwt and TTRV30M stay soluble unless treated with low-pH buffer [30], [31]. The complexes were immunoprecipitated with a SAP-specific antibody and the amount of SAP either bound to the aggregates or free in the remaining supernatants was determined. As shown Figure 1A, SAP co-incubated with pre-aggregated TTR also had the ability to bind pre-fibrillar aggregates of TTR formed *in vitro* at physiological pH by the TTR-D and TTR-A mutants. The state of these aggregates has been described in detail previously [30], [31]. Briefly, amorphous pre-fibrillar intermediates were formed, which transformed into mature fibrils similar in morphology to *ex vivo*-isolated material from the vitreous body. Immunoprecipitation using an anti-SAP antibody, followed by immunodetection with an anti-TTR antibody, revealed that SAP bound to TTR-D and to TTR-A in pre-fibrillar states and the complexes were found in the pellet, while TTRwt and TTR V30M remained in the supernatant fraction (Fig. 1A). Saturation binding measurements showed that the amount of SAP bound to aggregated TTR mutant proteins *in vitro* was low (7.5–8 µg SAP/mg TTR) compared to the amount bound to *ex vivo*-extracted vitreous amyloid fibrils (30 µg/mg). Still, these results are in the range (i.e. 5–20 µg SAP/mg dry weight amyloid fibril) previously reported by other researchers [19].

To exclude the possibility that SAP can interfere with aggregation of TTR in our experiments, we compared the migration pattern of TTR-A mutant subjected to *in vitro* aggregation at physiological pH for 0–5 days at 37°C with or without the presence of SAP. The aggregated material was analyzed further by native PAGE and detected with a monoclonal antibody that detects a cryptic epitope exposed only in the amyloidogenic form of TTR (residues 39–44 of the TTR sequence; [35]). We chose native PAGE to monitor the formation of TTR-A aggregates because this mutant is sensitive to low concentrations of SDS and dissociates into monomers–in contrast to TTRwt or TTRV30M, which form stable dimers (Fig. 1B). Remarkably, SAP neither promoted nor prevented aggregation of TTR-A mutant (Fig. 1C), demonstrated as no significant change in the migration pattern of aggregating TTR in the gels in the presence or absence of SAP. The starting material at day 0 migrated to the gel as a 50–60 kDa band corresponding to the size of tetramer, irrespective of the presence of SAP. Aggregates from incubation of TTR-A in 37°C after 1–3 days showed smears ranging from 100 to 250 kDa. In both the presence and absence of SAP, TTR-A showed indistinguishable time-dependent aggregation, apparent as an increase in high-molecular-weight aggregates. After 5 days, the TTR-A reached fibrillar state above 250 kDa and did not migrate into the separation gel.

### Effects of SAP on TTR-induced Toxicity

**Figure 2:**
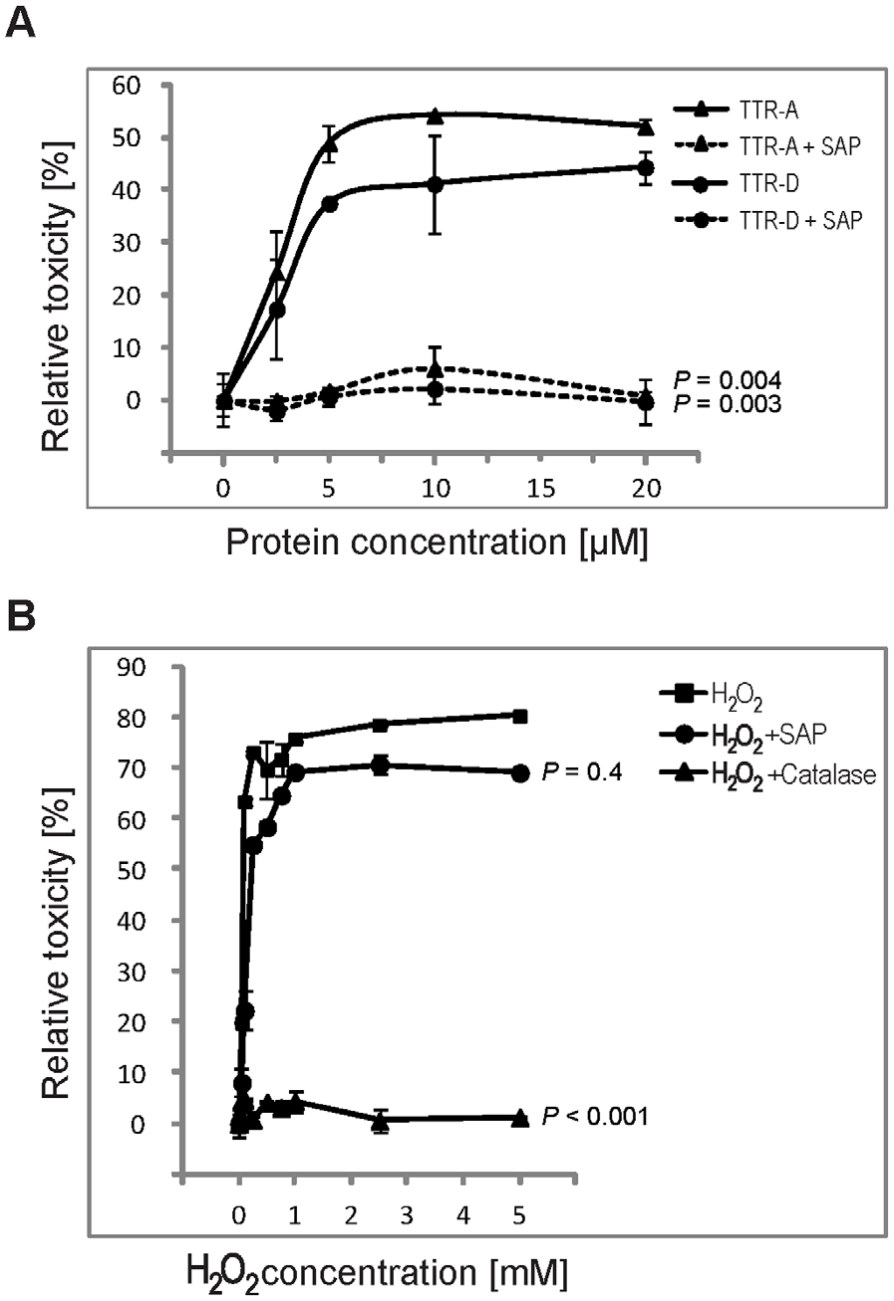
Effects of SAP on amyloidogenic aggregates. *(A)* The effect of SAP on TTR-induced toxicity. IMR-32 cells were incubated with the indicated concentrations of either TTR-A (▴) or TTR-D (•) for 12 h. Solid lines represent the toxic response when cells were incubated with the respective proteins, and dashed lines represent experiments with the addition of 3 µM SAP. One-way ANOVA with sequential Bonferroni *post-hoc* test revealed significant protective effects of SAP on cells in the presence of either TTR-A or TTR-D (*P = *0.004 and *P = *0.003, respectively) *(B)* The effect of SAP on H_2_O_2_-induced cytotoxicity. IMR-32 cells were treated with different concentrations of H_2_O_2_ (in the range 0–5 mM) without addition of (▪) or in the presence of 1,000 U/ml catalase (▴) or 3 µM SAP (•). Oxidative stress-induced toxicity in IMR-32 cells was significantly reduced by catalase treatment (*P*<0.001; one-way ANOVA, sequential Bonferroni *post-hoc* test) but not by SAP treatment (*P = *0.4). Error bars indicate SD.

Previous findings of cytotoxic effects associated with the pre-fibrillar aggregates of TTR, along with the present result on the binding of SAP to mutated pre-fibrillar TTRs, prompted us to investigate whether the association between SAP and toxic TTR aggregates might have functional consequences. We used the human neuroblastoma cell line IMR-32, which has been established as a model for studies of TTR toxicity, and WST-1 assay to measure cytotoxic effects. Cells in the medium without the addition of TTRs or SAP served as control and their viability was arbitrarily set at 100%, meaning no (i.e. 0%) toxicity. All toxicity data are shown as percentage change relative to control (for details of calculations, see Material and Methods). In the experiment, SAP was co-incubated with different concentrations of either TTR-A or TTR-D. After 12 h in culture, both mutants induced cell death in a dose-dependent manner with the maximum toxicity reached within the 5–20 µM range of TTR concentration. SAP at 3 µM totally inhibited the toxic response of the neuroblastoma cell line IMR-32 to pre-fibrillar aggregates of either TTR-A or TTR-D (Fig. 2A). Interestingly, this protective feature was unique to SAP since none of several other amyloid-associated molecules, i.e. hyaluronic acid, chondroitin sulfate A, B and C, or the pentraxin family member CRP, had any effect on the TTR-induced toxicity (Fig. S1).

It has been reported that amyloid toxicity is dependent on free radical production, and increased levels of H_2_O_2_ and lipid peroxides have been shown to accumulate in cells exposed to several amyloidogenic peptides [36], [37]. Antioxidants such as vitamin E or catalase, a potent scavenger of H_2_O_2_, have been shown previously to block both Aβ- and TTR-induced toxic responses in the IMR-32 cell line [34]. We therefore tested whether SAP can rescue IMR-32 cells from the oxidative stress induced by increasing doses of H_2_O_2_ (0–5 mM). The cells that were exposed to 0.05 mM H_2_O_2_ showed some toxic responses measured after 24 h with WST-1 assay; this toxic effect reached its maximum at H_2_O_2_ concentrations between 1 and 5 mM. When catalase (1,000 U/ml) was added to IMR-32 cells in the presence of H_2_O_2_, oxidative damage was blocked and the cells remained as metabolically active as in the controls without H_2_O_2,_ catalase, or SAP. Importantly, in contrast to the positive result obtained with catalase, we were unable to demonstrate that SAP could block H_2_O_2_-induced cell death, thus excluding SAP as an oxidative stress scavenger (Fig. 2B).

### SAP Prevents TTR-induced Toxicity

**Figure 3:**
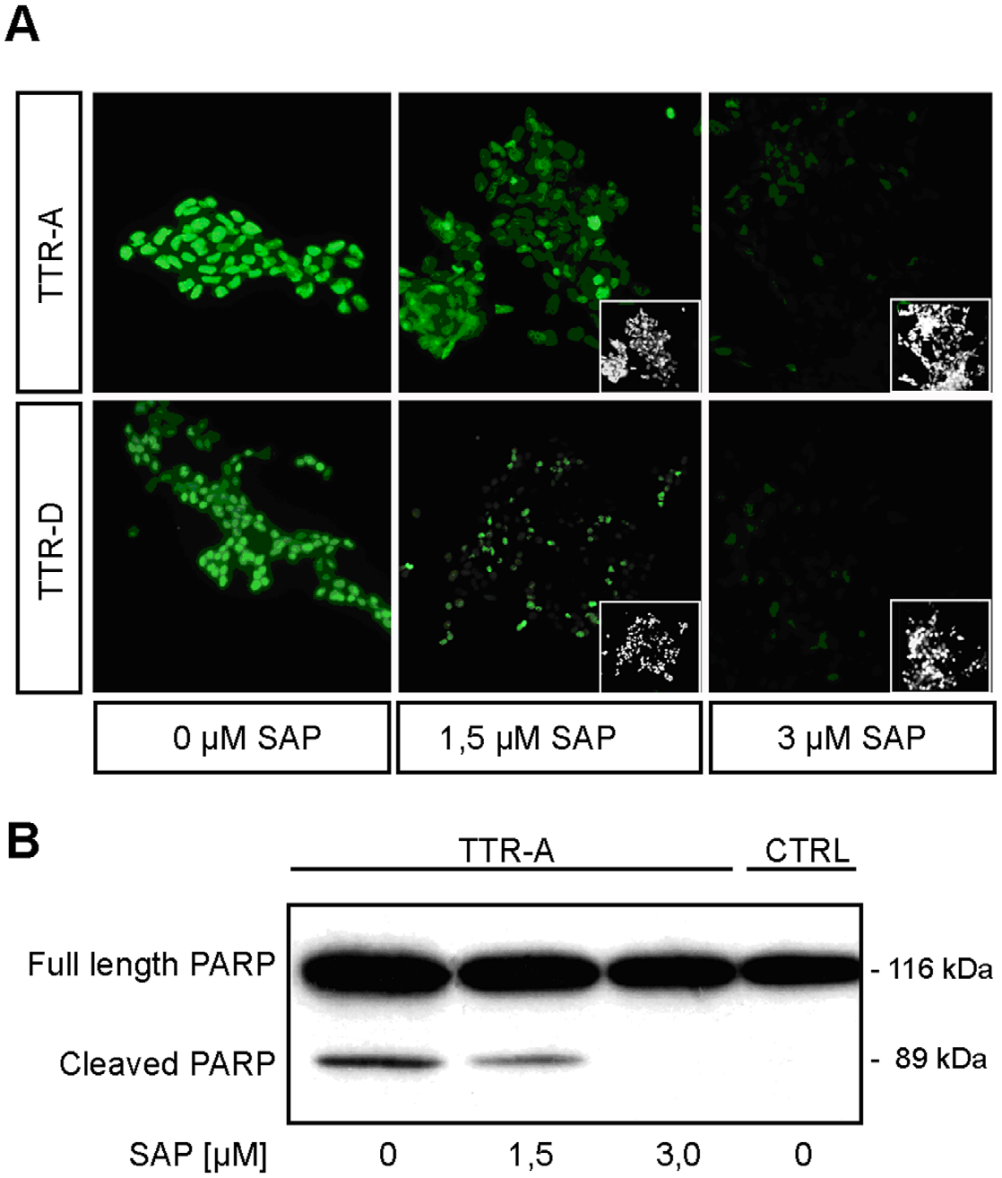
SAP prevents TTR-induced toxicity. *(A)* TUNEL staining of cells treated with amyloid protofibrils in the presence of SAP. IMR-32 cells were exposed to 20 µM TTR-A (upper row) or 20 µM TTR-D (lower row). The apoptotic response of the cells incubated with the amyloidogenic aggregates was assessed by TUNEL in the absence of SAP (left panel) or in the presence of either 1.5 µM SAP (middle panel) or 3 µM SAP (right panel). The inserts show the total amount of seeded cells in the slide chambers. *(B)* Analysis of PARP cleavage in TTR-treated and TTR/SAP-treated IMR-32 cells. Western blot analysis of PARP cleavage fragment (89 kDa) was performed with a PARP-specific antibody in extracts from cells treated with 20 µM TTR-A alone or in the presence of either 1.5 µM SAP or 3 µM SAP, and the results were compared against the negative control (untreated cells; CTRL). Full-length PARP has a molecular mass of 116 kDa.

The WST-1 assay used above does not provide any information on the type of cytotoxic response induced by mutated TTR. However, we have previously shown that both TTR-A- and TTR-D-induced toxicity is associated with apoptotic cell death. In order to study functional effects of SAP binding to pre-fibrillar aggregates, we used TUNEL (terminal deoxynucleotidyl transferase-mediated dUTP nick end-labeling) assay to visualize apoptosis. IMR-32 cells were exposed to 20 µM pre-aggregated TTR-A or TTR-D for 3 days, either alone or in the presence of 1.5 µM or 3 µM SAP. In Figure 3A, we present evidence that SAP prevented apoptosis caused by pre-fibrillar aggregates of mutated TTR. Exposure of IMR-32 cells to 20 µM TTR-A (upper row) or 20 µM TTR-D (lower row) induced TUNEL reactivity (green fluorescence), with almost all cells staining positive (left column). Premixing the amyloidogenic mutants of TTR with 1.5 µM SAP reduced the apoptotic cell response (middle column), while 3 µM SAP almost totally extinguished TUNEL-positive reactions (right column).

PARP (poly (ADP-ribose) polymerase) cleavage has been used as a marker for downstream effector caspases (Fig. 3B) [38]. The full length PARP (116 kDa) is involved in DNA repair mechanisms and helps cells to maintain their viability. Cleavage of PARP separates its carboxy-terminal catalytic domain (89 kDa) and facilitates cellular disassembly and apoptotic cell death. The IMR-32 cells were incubated for 12 h with 20 µM pre-aggregated mutated TTR-A in the presence or absence of SAP, and thereafter the cells were lysed with 0.1 M Tris, pH 6.8, 2% w/v SDS, and 1% v/v β-mercaptoethanol and analyzed by SDS-PAGE. Figure 3B shows that there was a clear cleavage of PARP in cell lysates after co-incubatation with pre-fibrillar TTR-A, with fragments of the expected sizes. When TTR-A was mixed with 1.5 µM SAP, a clear reduction in the cleavage was observed, while in the presence of 3 µM SAP no traceable fragments of PARP were seen–similarly to the control IMR-32 cells that were treated with neither TTR-A nor SAP.

### Co-expression of SAP and TTR-A in *Drosophila* Protects from Development of the Dragged-wing Phenotype

**Figure 4:**
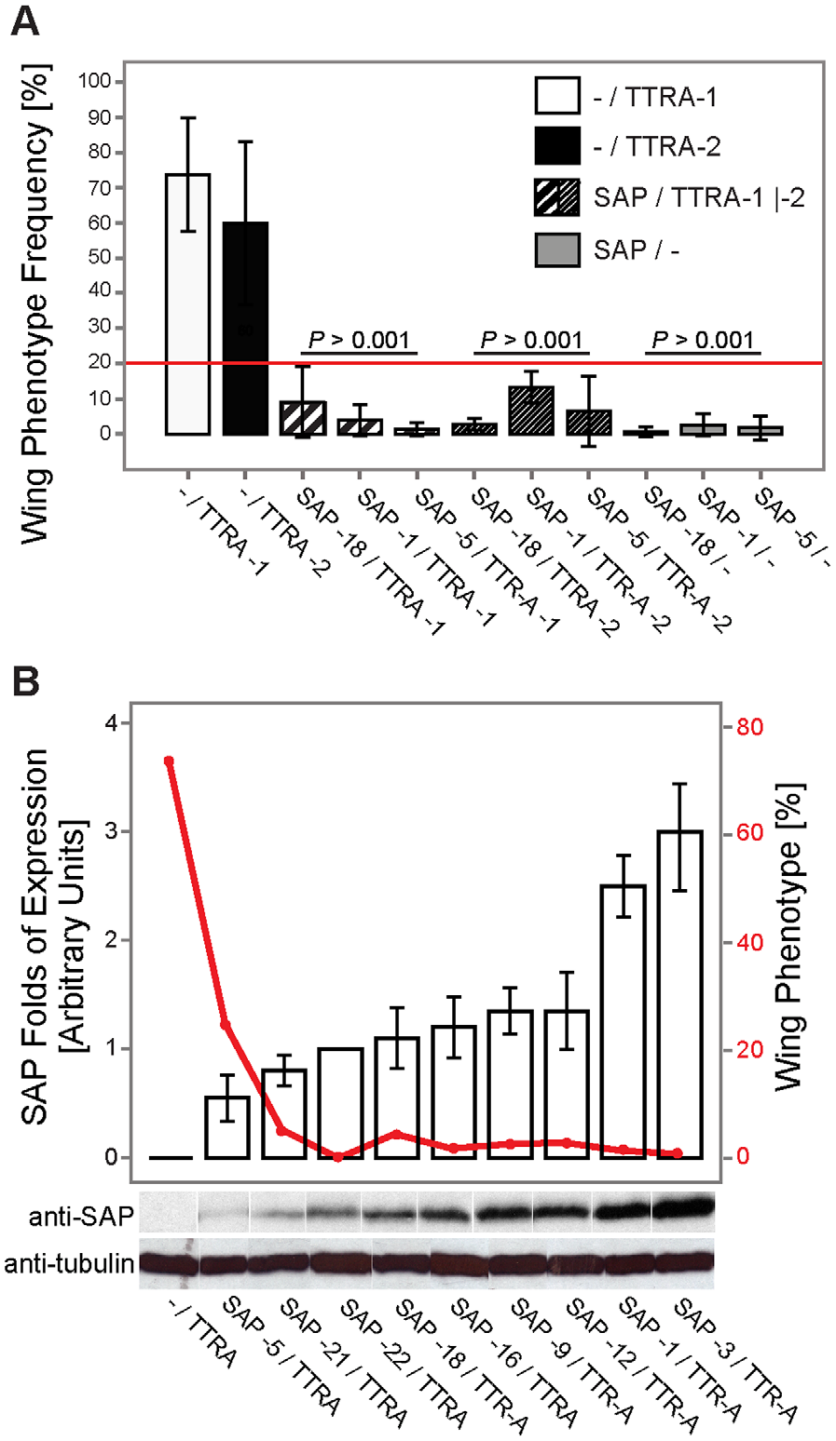
Dose-dependent reduction of the frequency of the dragged-wing phenotype in fruit flies that co-expressed TTR-A and SAP. *(A)* Two independent TTR-A transgenic strains (*w; GMR-Gal4/+; UAS-TTR-A/+* designated TTRA-1 presented as white bars, and TTRA-2 as black bars) and three independent SAP-transgenic strains (*w; GMR-Gal4/+; UAS-SAP/+;* SAP-18, SAP-1 and SAP-5, represented by gray bars)–either alone or in combination with each other (black-dashed white bars)–were analyzed for occurrence of the dragged-wing posture (mean values; error bars indicate SD). Significant reduction in the frequency of abnormal wings (below 20%, red line) was observed upon co-expression of SAP in both strains of TTR-A (*P<*0.001 for all SAP/TTR-A and SAP/− vs. TTR-A genotypes; one-way ANOVA, sequential Bonferroni *post-hoc* test). *(B)* Dose-dependent reduction in the frequency of the dragged-wing phenotype in flies expressing both TTR-A and SAP. SAP had a significant protective effect against TTR-A toxicity, as seen from the mean value of the wing phenotype (descending red line). Expression levels of SAP (white bars) were quantified in nine independent UAS-SAP-transgenic lines and are presented as the fold change in relation to tubulin levels (mean values; error bars indicate SD). Representative immunoblots are shown in the panel below the diagram.

Soon after eclosure, *Drosophila melanogaster* overexpressing the secreted form of TTR-A, but not wild-type TTR, develops the dragged-wing phenotype [32]. This early phenotype reflected the overall state of toxic TTR-A formed in fruit flies and correlated well with other TTR-A-induced phenotypes such as neurodegeneration, locomotor dysfunction, and premature death. In the experiment (Fig. 4A), we used two independent transgenic lines with a single copy of the TTR-A gene (designated TTRA-1 and TTRA-2) that showed variable frequency of abnormal wings (∼60–74% ±25%). Figure 4A demonstrates a significant protective effect of SAP co-expression (in four independent SAP-expressing transgenic strains) on the TTR-induced phenotype, seen as a reduction in dragged-wing posture (below 20%, red line) to almost complete rescue (∼1.3%). Overexpression of SAP on its own in these strains did not lead to any noticeable alterations in wing position. The protection against TTR-A toxicity by SAP was dose-dependent, as increased levels of SAP expression (normalized against tubulin levels; represented by bars in Fig. 4B) lowered the frequency of dragged wings (solid line in Fig. 4B).

### SAP Co-localizes with TTR-A in *Drosophila* Eye and Prevents Retinal Degeneration

**Figure 5:**
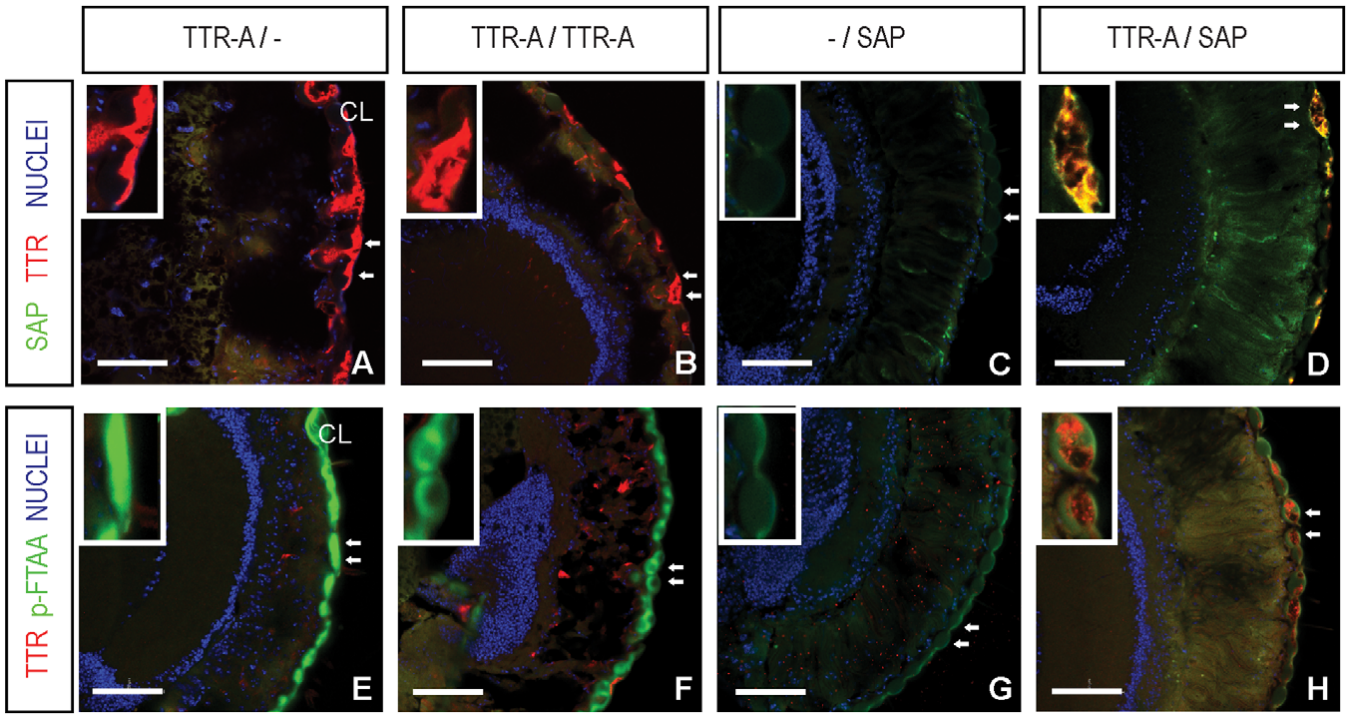
SAP co-localizes with TTR-A in *Drosophila* eye and counteracts TTR-induced retinal degeneration. *(A–D)* TTR-A was detected with a TTR-specific monoclonal antibody (Mab39–44; in red) [35], [58], and co-localized with SAP immunostaining (Epitomics; in green) in horizontal sections of heads of 2-week-old flies. *(E–H)* TTR-A was detected with TTR-specific polyclonal antibody (red). TTR-A aggregates were monitored with p-FTAA (green). (*A, B*) TTR-A secreted by the photoreceptors accumulated in the retinal compartment (*E, F*) and formed aggregates around the outer corneal layer (CL). This led to damage of the retinal array and leakage of TTR-A outside the CL. The two neighboring corneal lenses (arrows) are shown magnified at the upper left corner (insets). (*C*) SAP expressed alone in fly retina stayed soluble, as no p-FTAA aggregates were detected (*G*) and there were no degenerative changes. Co-localization of SAP with TTR-A prevented retinal damage in SAP/TTR-A fruit flies (*D*), and led to reduced p-FTAA staining in the CL (*H*). *Drosophila* genotypes: TTR-A/− (*w; GMR-Gal4/+; UAS-TTR-A/+*); TTR-A/TTR-A (*w; GMR-Gal4/GMR-Gal4; UAS-TTR-A/UAS-TTR-A*); −/SAP (*w; GMR-Gal4/+; +/UAS-SAP*); TTR-A/SAP (*w; GMR-Gal4/+; UAS-SAP/TTR-A*). Scale bar represents 50 µm.

To monitor SAP and TTR-A interaction further, we performed immunohistochemical analysis of fly retinas that co-expressed these two proteins (Fig. 5). TTR-A expressed alone leads to retinal degeneration, as reported previously [32], [33]. In Figure 5, we show that in 2-week-old flies, TTR-A secreted by the photoreceptors (Fig. 5A and B, red) accumulated as amyloid aggregates in the retinal compartment around the outer corneal layer (CL), which was confirmed with the amyloid-specific dye p-FTAA [39], [40] (Fig. 5E and F, aggregated TTR-A in green). This resulted in damage to the retinal array and leakage of TTR-A outside the CL (individual corneal lenses shown with arrows are magnified in the insets in Fig. 5). In contrast, SAP expressed alone in the fruit fly retina stayed soluble (Fig. 5C), as no p-FTAA aggregates were detected (Fig. 5G) and no degeneration of the retina was observed compared to control flies (Fig. S2A). Co-expression of SAP and TTR-A (Fig. 5D and H) resulted in complete protection from the degenerative changes induced by TTR-A. Interestingly, SAP co-localized with TTR-A aggregates, as clearly seen in Figure 5 D (additional details in Fig. S2B and C) and by reduced p-FTAA staining of TTR-A (Fig. 5H).

Immunoblot analysis of non-reduced head extracts confirmed co-localization of SAP with aggregated TTR-A in fruit flies co-expressing these two proteins (Fig. S3). These flies showed some levels of soluble TTR-A and of unbound monomeric SAP, and had normal wing posture. In contrast, in flies that only expressed TTR-A and that had the dragged-wing phenotype, no soluble TTR-A was detected, as TTR formed large aggregates that did not enter the gel.

## Discussion

The physiological significance of SAP is not well understood. No deficiency state has been reported in any mammalian species, which indicates that it has an important conserved physiological function. A number of biological properties have been suggested, some of which are contradictory. The highly specific binding of SAP to nuclear chromatin, *in vitro* and *in vivo*, and the solubilizing effect of this interaction on the otherwise insoluble chromatin may be functionally important. It has been suggested that SAP prevents an autoimmune reaction by binding to free chromatin, although this has been disputed [41]. There is as yet no known biophysical basis for why SAP binds to such structurally different molecules as DNA, histones, and LPS.

Amyloid formation with similar structure and similar toxic propensities appears to be an inherent property of the amyloidogenic proteins [42]. SAP binds to most types of amyloid fibrils *in vivo*, to fibrils extracted *ex vivo*, and to fibrils formed from pure proteins or peptides *in vitro*, suggesting interaction with a structural motif that is common to all amyloid fibrils. It has been suggested that decoration of amyloid fibrils with SAP prevents the fibrils from degradation by proteases [43]. Contradictory results have been published concerning the ability of SAP to promote and to prevent Aβ aggregation [22], [23]. Our finding that *in vitro* aggregation of TTR is not affected by SAP supports the notion that SAP is dispensable for the formation of amyloid fibers (Fig. 1C). Furthermore, induction of SAP synthesis in transgenic mice does not appear to affect the onset and extent of TTR deposition [25].

We have previously described a toxic response induced by protofibrils, but not by mature amyloid [34], which now appears to be the common mechanism for most amyloid-forming proteins [44], [45]. In this study, we were interested in seeing whether SAP could affect TTR-induced cytotoxicity, and we were able to show that SAP is the only amyloid-associated molecule of those tested that has an inhibitory effect on the toxic response caused by mutated TTR.

The mechanism of amyloid-induced cytotoxicity is not clear. One possibility is through generation of free radicals, as catalase was able to prevent the toxic response [36]. This is further supported by studies on the role of lipid peroxidation products surrounding both TTR and Alzheimer’s amyloid deposits [46], [47]. Treatment of cells with graded doses of hydrogen peroxide and different concentrations of SAP did not support the existence of such a mechanism in this study.

Interestingly, we found that SAP prevented apoptosis in a dose-dependent way, as measured by TUNEL response and PARP cleavage. The SAP concentrations chosen (Figure 3) were within the physiological range and clearly inhibited toxicity caused by aggregating mutant TTR in molecular excess. These data add to previous findings on protection from noxious protein aggregates. Thus, we and others have shown that maturation from pre-fibrillar aggregates to amyloid might represent a rescue mechanism [29], [34], [48]. Amyloid may represent a thermodynamic trap and appears to be a generic protein conformation [45], [49], which is an important mechanism for avoidance of unstable toxic peptide aggregates. We propose that decoration of amyloid fibrils and their pre-aggregated precursor states with SAP could be another defense mechanism against formation of toxic aggregates.

Taken together, our data suggest that there is an interaction between SAP and pre-fibrillar aggregates, which results in block of apoptosis. Characterization of the molecular mechanisms awaits two important missing details: (1) the nature of the mechanism of induction of toxicity by pre-fibrillar aggregates, and (2) the nature of the biophysical interaction between SAP and the toxic aggregates. Before these questions are answered, we cannot propose a mechanism to explain our observations.

To confirm that SAP has a role in the pathogenesis of TTR-associated amyloidosis, we performed a genetic study in transgenic *Drosophila melanogaster*. Co-expression of SAP and TTR-A in the fruit fly confirmed our *in vitro* findings regarding the protective role of SAP in TTR-induced toxicity. We were able to show that molecular interaction between SAP and TTR-A counteracts the deleterious effects of TTR-A aggregation, manifested as the dragged-wing phenotype and retinal degeneration in TTR-A flies. The effect was dose-dependent in both *in vitro* and *in vivo* studies. It is important to investigate the mechanism behind SAP-TTR interaction in greater detail, since SAP may have an important modulatory role in TTR-associated amyloidosis. We propose that SAP recognizes and binds to early toxic aggregates of TTR and thus constrains the toxic effects of the pre-fibrillar species of TTR.

The possible role of SAP as an inhibitor of protease-aided amyloid breakdown has been exploited for drug development. Preclinical evaluations of a competitive inhibitor of SAP binding to amyloid fibrils have shown depletion of SAP in plasma, with resulting regression of systemic visceral amyloid deposits [50], [51]. However, amyloid might cause harm through several different mechanisms. Massive deposits, as seen in lysozyme-associated amyloidosis or certain forms of TTR-associated amyloidosis, could be deleterious due to the volume–or to mechanical effects on heart movements, for example. A more biochemical mechanism that has been proposed highlights the importance of small oligomeric aggregates formed early in or off the fibrillogenesis pathway as the main mediators of pathogenicity [45]. Toxic species of TTR have been identified both in *ex vivo* explants from patients and *in vitro* and *in vivo* models, including the fruit fly [29], [32]–[34], [52]. Despite the fact that the structure of different amyloids is well known, there is no evidence for a correlation between the extent of final deposits and severity of the disease [53], [54]. Previous findings have shown that early TTR aggregates bind cellular receptors [55] and cause harm without the presence of visible fibrillar amyloid deposits [52], [56]. We propose that the approach with SAP inhibitors should be handled with caution in the early stages of fibril formation, since SAP might reduce the toxic effects. In the later stages of the disease, with excessive deposits, this approach could be beneficial by reducing the size–and therefore the adverse (mechanical) effects–of amyloid load.

## Material and Methods

### Ethics Statement

SAP was purified from human plasma, which was obtained from outdated blood donations from the local blood bank (Blodcentralen Umeå; Department of Clinical Immunology and Transfusion Medicine, Umeå University Hospital, SE-901 85 Umeå, Sweden) and only from anonymous donors, precluding the need for informed consent. According to Swedish law (the Ethical Review Act from 2004), ethical review is only necessary when the personal integrity of identifiable individuals is under threat.

### Purification of SAP

SAP was purified from human plasma according to Anderson and Mole [57], with slight modifications. No BaCl_2_ precipitation was done prior to ammonium sulfate treatment. The purified protein was stored in 0.01 M Tris, pH 8, 0.14 M NaCl, and 10 mM EDTA. Prior to use, SAP was diluted in 0.01 M Tris, pH 8, 0.14 M NaCl, 5 mM Ca^2+^, and 0.3% human serum albumin (HSA) to the concentrations indicated.

### Purification of Recombinant TTR Mutants

pET3a plasmids with TTR inserts were used to transform competent *E. coli* BL21 cells for expression of TTR protein as previously described [30]. Both the TTR-D (G53S/E54D/L55S) and the TTR-A (V14N/V16E) mutants formed inclusion bodies in *E. coli*; these were purified as described [30], [31]. Briefly, the cells were lysed with lysozyme (1 mg/ml) for 30 min at room temperature and treated with DNase in the presence of 20 mM Mn^2+^ ions until the solution was no longer viscous. The inclusion bodies were washed in buffer (50 mM Tris-HCl, pH 7.5, and 1 mM EDTA) and collected by centrifugation. The pellet was solubilized (0.01 M phosphate buffer, pH 7.2, 4 M urea, and 1% v/v β-mercaptoethanol) and the remaining insoluble material was pelleted and discarded. After dialysis overnight at 4°C against deionized water, the material was run on a DEAE-Sepharose FF column in 0.05 M Tris-HCl, pH 7.5, and eluted with a linear NaCl gradient (0–0.5 M). The main protein peak was collected, which contained a single protein band of 16 kDa in SDS-PAGE, which was confirmed to be TTR by immunoblotting. Wild-type TTR or TTV30M did not form inclusion bodies. This allowed the supernatant obtained after lysis of the cells to be directly dialyzed overnight against 50 mM Tris-HCl, pH 7.5, and 0.05 M NaCl, and run on a DEAE-Sepharose FF column as described above.

### Aggregation of TTR

Prior to use, all proteins were diluted in 0.01 M Tris, pH 8, 0.14 M NaCl, 5 mM Ca^2+^, and 0.3% HSA, and incubated at 37°C for the indicated time periods to allow generation of pre-fibrillar aggregates of TTR [34].

### Source of Reagents

Heparan, hyaluronic acid, chondroitin sulfate A, B, and C, catalase, and hydrogen peroxide (H_2_O_2_) were all purchased from Sigma-Aldrich, Sweden.

### Binding of SAP to TTR in ELISA

Binding of SAP to pre-aggregated TTR was performed according to the protocol by [19] with some modifications. Aliquots of 10 µg pre-aggregated recombinant TTRs were mixed with different concentrations of SAP (0–300 ng/ml) in 0.01 M Tris-buffered NaCl (0.138 M) containing 0.005 M CaCl_2_, pH 8.0, and incubated at room temperature for 90 min. After this incubation, the protein material was spun down and the supernatants were collected before washing the remaining material with fresh incubation buffer. Bound SAP was extracted from the fibrils using EDTA containing buffer (0.01 M Tris, pH 8.0, 0.14 M NaCl, and 10 mM EDTA). Soluble SAP in all the supernatants (before and after EDTA extraction) was measured in a sandwich ELISA using NUNC 96-well microtiter plates coated with a rabbit polyclonal antibody raised against human SAP (DAKO, Glostrup, Denmark) at a concentration of 5 µg/ml in phosphate-buffered saline (PBS). Detection was performed with a rabbit polyclonal horseradish peroxidase-labeled antibody raised against human SAP (DAKO) as described previously [35], [58]. Vitreous eye amyloid fibrils from a patient with the V30M TTR mutation were prepared as described previously [35], and suspended in Tris-buffered saline containing CaCl_2_, pH 8.0.

### Immunoprecipitation and Immunoblotting

Prior to binding, aliquots of 10 µg recombinant TTRs were pre-aggregated into protofibrils at 37°C for 4–6 days. TTRs and SAP (35 ng/µg of TTR) were mixed in 0.5 ml calcium-containing Tris-buffered saline (10 mM Tris, pH 8.0, 0.14 M NaCl, 0.005M CaCl_2_, and 4% w/v BSA) and incubated at room temperature for 12 h. The complexes were immunoprecipitated with a SAP-specific antibody (DAKO) coupled to magnetic beads (Dynabeads M-280; Dynal, Norway) according to the manufacturer. Both the pellet and the supernatant were resolved on 15% (w/v) SDS-PAGE. Proteins were transferred to a PVDF-plus membrane as described before [34]. Blocking was performed with 5% w/v skimmed milk with 0.05% v/v Tween-20. Immunodetection was performed with a polyclonal rabbit anti-human TTR antibody (1∶2,000; DAKO, Sweden), followed by a secondary horseradish peroxidase-labeled donkey anti-rabbit IgG antibody (Amersham Pharmacia Biotech, Uppsala, Sweden). The immunoreaction was detected with SuperSignal® Substrate (PIERCE, Rockford, IL) according to the manufacturer. In the experiment where we analyzed TTR-A aggregate formation by 10% native PAGE, the protein material was mixed with a native PAGE loading buffer (0.1 M Tris, pH 6.8, without any β-mercaptoethanol or SDS) and loaded onto the gels. Electrophoresis was run at 120 V at 4°C for 1.5 h. The proteins were transferred in a semi-dry blot apparatus onto PVDF membrane with Towbin buffer (0.025 M Tris, 0.192 M glycine, and 20% v/v methanol). Immunodetection was performed with a monoclonal mouse anti-human amyloidogenic TTR antibody (positions 39–44 of the TTR sequence; [35]), followed by a secondary horseradish peroxidase-labeled goat anti-mouse IgG antibody (Amersham Pharmacia Biotech, Uppsala, Sweden). The immunoreaction was detected as above.

### Cell Culture

The cell line IMR-32 (human neuroblastoma) was obtained from Professor Sven Påhlman, Lund University, Sweden [59]. The cells were grown in Iscove’s medium, supplemented with 10% (v/v) FBS, 1% v/v non-essential amino acid solution, soybean lipids, 0.1% (v/v) ITS-G, 3% (w/v) HSA, and 100 units/ml PEST (Gibco Cell Culture; Invitrogen, Sweden) at 37°C in a humidified atmosphere of 5% (v/v) CO_2_ in air. The cells were seeded at a density of 10,000 per well in 96-well microtiter plates (Falcon; Becton-Dickinson, NJ).

### Determination of Cytotoxic Activity

The cytotoxicity of TTR-A or TTR-D in the concentration range 0–20 µM and the effects of amyloid-associated molecules (0–3 µM) were assayed after 12 h in cultured IMR-32 cells using the WST-1 kit according to the manufacturer (Roche Diagnostics, Indianapolis, IN). The stable tetrazolium salt WST-1 (pink-colored) is converted to a soluble formazan (orange-colored) by viable cells. Cells were incubated with the WST-1 reagent for 2 h at 37°C in a humidified atmosphere of 5% (v/v) CO_2_ in air. After this period, formation of the formazan dye was quantified as absorbance (at 450 nm for maximum formazan absorption and 690 nm as reference wavelength) with a scanning multiwell spectrophotometer (ELISA reader). All absorbance data were used first to calculate relative cell viability by dividing the absorbance of a sample by the absorbance of the control.

Cells in medium without TTR aggregates or SAP served as controls, and their viability (equal to 1) was defined as no toxicity (0%) according to the formula: (sample viability –1)×(−1). All results are shown as the relative toxicity in relation to the controls and they are presented as mean values ± SD. All experiments were performed in duplicate and were repeated at least three times.

### TUNEL Assay

IMR-32 cells were cultured in 8-well Permanox slides (Lab-Tek Brand Products, Nalge Nunc International, Naperville, IL) at a density 2×10,000 cells/well as described previously [34]. Pre-aggregated TTR-A or TTR-D (20 µM), pre-incubated with or without 0–3 µM SAP, was incubated further with the cells for 12 h at 37°C in a humidified atmosphere of 5% (v/v) CO_2_ in air, and then TUNEL staining was performed according to the directions of the manufacturer (Roche Diagnostics).

### PARP Assay

Cell lysates were prepared from IMR-32 cells cultivated as described above for TUNEL assay, boiled in buffer containing 0.1 M Tris, pH 6.8, 2% w/v SDS, 2% v/v β-mercaptoethanol, and separated on 15% SDS-PAGE. The proteins were transferred to a PVDF-plus membrane (Micron Separation, Westboro, MA). The membrane was blocked with 5% (w/v) skimmed milk. Immunodetection was done with a polyclonal rabbit anti-PARP antibody that detected both full-length and cleaved fragment of human poly (ADP-ribose) polymerase (1∶2,000; #9542, Cell Signalling; In Vitro, Stockholm, Sweden), followed by a secondary horseradish peroxidase-labeled donkey anti-rabbit IgG antibody (Amersham Pharmacia Biotech, Uppsala, Sweden). The immunoreaction was detected with SuperSignal® Substrate (PIERCE, Rockford, IL) according to the manufacturer’s instructions.

### Transgenic Constructs and *Drosophila* Stocks

For the SAP construct, the relevant part of the human *APCS* gene with the endogenous signal sequence, using a cDNA clone (I.M.A.G.E. ID 3934872; Geneservice Ltd, Cambridge, UK, www.geneservice.co.uk) was amplified with HotMaster™ Taq DNA polymerase (Eppendorf) using the primers 5′-ACCCATATGGAATTCAATATGAACAAGCCGCTGCTTTGGATCTCTG-3′ and 5′-GGAGGATCCTCTAGAACCTCAGACCCACACCAAGGGTTTGATGAT-3′. Annealing was at 62°C for 10s and extension was at 72°C for 45s, for 30 cycles. An *Eco*RI−/*Xba*I-digested PCR fragment was cloned into pUAST-vector and its identity confirmed by DNA sequencing. Germ-line transformants were generated by standard procedures [60]. Nine independent *UAS-SAP* transgenic lines with differential levels of SAP expression (and no endogenous SAP) were used in the study (marked SAP-1, -3, -5, -9, -12, -16, -18, and -22 accordingly). The eye-specific driver w^*^; P{w ^+*mC*^ = *GAL4-ninaE.GMR}12*, abbreviated *GMR-Gal4* [61], was used and the *UAS-TTR* transgenic stocks have been described previously [32]. Expressing lines were generated using standard mating schemes. All flies were fed on standard mashed-potato/yeast/agar medium at 25°C and held under 12/12-h cycles of light and darkness.

### Assessment of Wing Phenotype

Wing posture was assessed in female flies soon after eclosure by counting dragged wings in populations of at least 100 flies per genotype, in three independent experiments. Data are given as mean values ± SD. Two independent *UAS-TTR-A* transgenic strains and nine independent *UAS-SAP*-transgenic strains were evaluated.

### Quantification of SAP Expression in Transgenic *Drosophila*

Heads of ten female flies were homogenized in 200 µl lysis buffer (50 mM Tris-HCl, pH 7.5, and 10% SDS (v/v) with complete protease inhibitors (Roche Diagnostics GmbH, Mannheim, Germany)) at 4°C, sonicated, boiled for 10 min at 100°C, and centrifuged for 10 min at 16,000×*g* (Eppendorf Microcentrifuge 5415C) at 4°C. Total protein concentrations in supernatants of fruit fly extracts were estimated with the BCA Protein Assay (PIERCE). Ten µg of total protein was resolved on 15% Criterion Tris-HCl precast gels (Bio-Rad Laboratories) and transferred electrophoretically onto Hybond-C Extra nitrocellulose membrane (0.45 µm pore size; Amersham Biosciences). Immunodetection was performed with either a monoclonal rabbit anti-human SAP antibody (1∶5,000; Cat. no. 1793-1; Epitomics, Burlingame, CA) or a monoclonal mouse anti-α-tubulin (1∶8,000; T8203; Sigma, Saint Louis, MO), followed by a secondary horseradish peroxidase-labeled goat anti-rabbit or goat anti-mouse IgG antibody (1∶10,000; Amersham Pharmacia Biotech, Uppsala, Sweden), respectively. Expression levels of proteins were quantified by detection of chemiluminescence (SuperSignal® West Pico Chemiluminescent Substrate; PIERCE) using a Fluor-S Multi-Imager with Quantity One software (Bio-Rad, Richmond, CA). Expression of SAP is presented as fold change in relation to tubulin levels and was calculated as mean values ± SD from two independent experiments.

### Immunohistochemistry and Fluorescence Microscopy

Semi-thin cryosections (10 µm) of heads from 2-week-old flies were fixed with 4% (w/v) formaldehyde in PBS, pH 7.3, and incubated in 10% (v/v) normal horse serum in blocking buffer (0.3% Triton X-100 in PBS). The primary antibodies used were mouse monoclonal anti-TTR (Mab 39–44, 1 µg/ml, [30], [58]), rabbit monoclonal anti-SAP (1∶200; Cat. no. 1793-1; Epitomics) or rabbit polyclonal anti-TTR (1∶300, pre-adsorbed; A0002; DAKO). As secondary antibodies we used Rhodamine Red-X-AffiniPure goat anti-mouse IgG or FITC-AffiniPure goat anti-rabbit IgG (1∶250; Jackson ImmunoResearch Laboratories, Inc., West Grove, PA). p-FTAA was used to stain for amyloid aggregates [39], [40]. All specimens were mounted on slides with VECTASHIELD mounting medium containing DAPI to counterstain nuclei (Vector Laboratories, Burlingame, CA). Fluorescence images were obtained with a Zeiss LSM 710 confocal microscope with Zen 2009 Light Edition software (Zeiss GmbH, Jena, Germany) and they were assembled using Adobe Photoshop and Illustrator CS4 (Adobe Systems Inc., San Jose, CA).

### Statistical Analysis

Statistical analysis was performed using PASW Statistics 18.0 for Windows (IBM Corporation, Armonk, NY). We used one-way ANOVA with the Bonferroni correction for multiple comparisons. The mean difference was considered to be statistically significant at the 95% confidence level.

## Supporting Information

Figure S1 **Effect of pentraxins and glycosaminoglycans on TTR-induced toxicity.** IMR-32 cells were incubated with the indicated amounts of amyloid-associated molecules such as CRP, hyaluronic acid, chondroitin sulfate A, B and C (solid lines within the dashed area), in the presence of 20 µM TTR-A mutant. These amyloid-associated molecules showed no reduction of TTR-A-induced cytotoxicity. SAP was the only protective molecule that had a distinct effect on TTR-induced cell death (•). TTR toxicity was measured by WST assay described in Materials and Methods and presented as mean values of relative toxicity (%) ± SD.
(PDF)

Figure S2 **Colocalization of SAP (green) and TTR-A (red) in 2-week-old fly head horizontal cryosections.** Nuclei were counter-stained with DAPI (blue). (***A***) In contrast to the control fly retina, where no TTR was detected, (***B***) in TTR-A expressing flies TTR-A secreted by the photoreceptors accumulated in the retinal compartment and formed aggregates (red spots) around the outer corneal layer (CL). This led to damage of the retinal array and leakage of TTR-A outside the CL. Individual corneal lenses shown with arrows are magnified in the figure insets. (***C***) Colocalization of SAP with TTR-A prevented retinal damage in SAP/TTR-A flies. (***A***) Control fly (*w; +/+; +/+*), (***B***) TTR-A/− (*w; GMR-Gal4/+; UAS-TTR-A/+*), (***C***) TTR-A/SAP (*w; GMR-Gal4/+; UAS-SAP/TTR-A*). Scale bar represents 50 µm.
(PDF)

Figure S3 **Immunodetection of SAP and TTR-A in non-reduced fly head extracts.** SAP binding to TTR-A was compared between flies co-expressing SAP and TTR-A (*w; GMR-Gal4/+; UAS-SAP/UAS-TTR-A*) or TTR-A alone (*w; GMR-Gal4/+; UAS-TTR-A/+*) in two independent experiments. It confirmed colocalization of SAP with aggregated TTR-A (250 kDa, HMW, High Molecular Weight aggregates) in flies co-expressing these two proteins. Flies that had normal wing posture, showed some levels of soluble TTR-A as well as unbound monomeric SAP. In contrast, in TTR-A only expressing flies with the dragged wings, TTR aggregated and did not enter the gel as no soluble TTR-A was detected. Two independent transgenic lines of SAP expressing flies were used in the analysis denoted SAP-18 and SAP-3.
(PDF)
