# Supplementary figures and images for: Inhibition of TTR Aggregation-Induced Cell Death – A New Role for Serum Amyloid P Component
Source: PLoS One. 2013 Feb 4;8(2):e55766. doi: 10.1371/journal.pone.0055766 (PMC3563535; doi:10.1371/journal.pone.0055766)

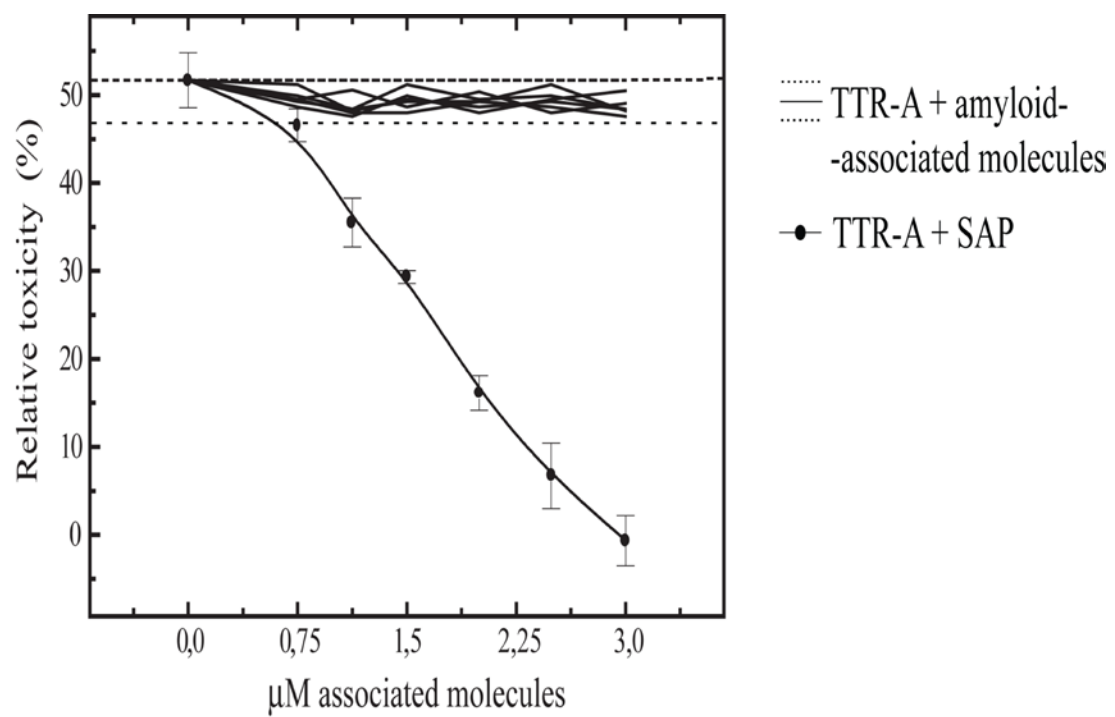

Supplement: Figure S1 — Effect of pentraxins and glycosaminoglycans on TTR-induced toxicity. IMR-32 cells were incubated with the indicated amounts of amyloid-associated molecules such as CRP, hyaluronic acid, chondroitin sulfate A, B and C (solid lines within the dashed area), in the presence of 20 µM TTR-A mutant. These amyloid-associated molecules showed no reduction of TTR-A-induced cytotoxicity. SAP was the only protective molecule that had a distinct effect on TTR-induced cell death (•). TTR toxicity was measured by WST assay described in Materials and Methods and presented as mean values of relative toxicity (%) ± SD. (PDF) [file pone.0055766.s001.pdf]

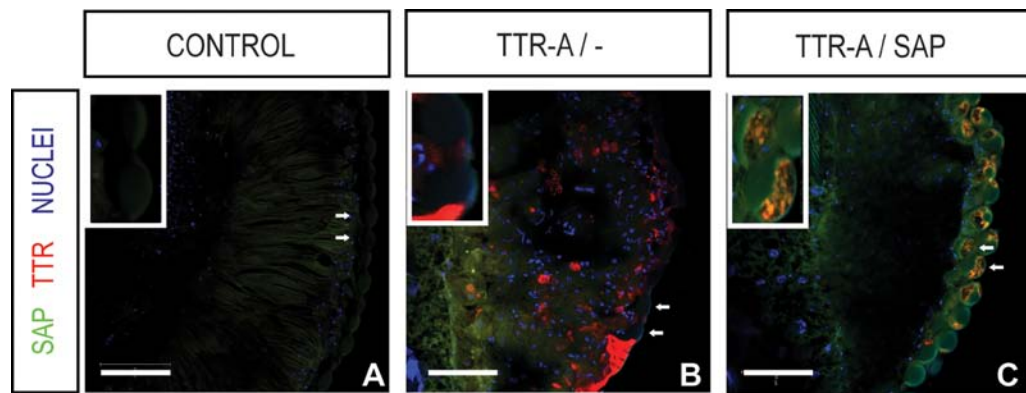

Supplement: Figure S2 — Colocalization of SAP (green) and TTR-A (red) in 2-week-old fly head horizontal cryosections. Nuclei were counter-stained with DAPI (blue). (A) In contrast to the control fly retina, where no TTR was detected, (B) in TTR-A expressing flies TTR-A secreted by the photoreceptors accumulated in the retinal compartment and formed aggregates (red spots) around the outer corneal layer (CL). This led to damage of the retinal array and leakage of TTR-A outside the CL. Individual corneal lenses shown with arrows are magnified in the figure insets. (C) Colocalization of SAP with TTR-A prevented retinal damage in SAP/TTR-A flies. (A) Control fly (w; +/+; +/+), (B) TTR-A/− (w; GMR-Gal4/+; UAS-TTR-A/+), (C) TTR-A/SAP (w; GMR-Gal4/+; UAS-SAP/TTR-A). Scale bar represents 50 µm. (PDF) [file pone.0055766.s002.pdf]

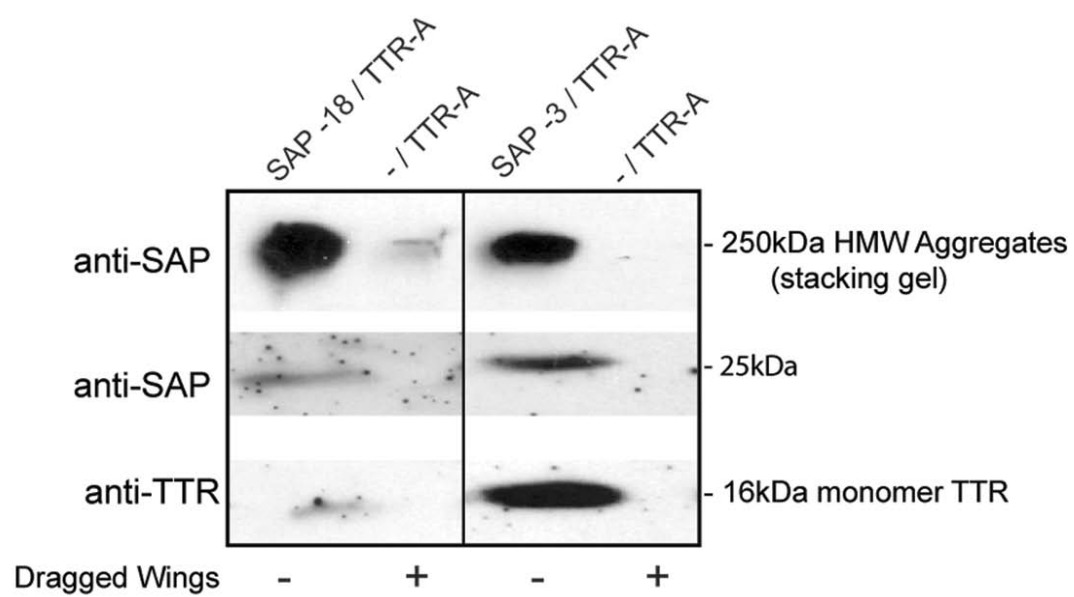

Supplement: Figure S3 — Immunodetection of SAP and TTR-A in non-reduced fly head extracts. SAP binding to TTR-A was compared between flies co-expressing SAP and TTR-A (w; GMR-Gal4/+; UAS-SAP/UAS-TTR-A) or TTR-A alone (w; GMR-Gal4/+; UAS-TTR-A/+) in two independent experiments. It confirmed colocalization of SAP with aggregated TTR-A (250 kDa, HMW, High Molecular Weight aggregates) in flies co-expressing these two proteins. Flies that had normal wing posture, showed some levels of soluble TTR-A as well as unbound monomeric SAP. In contrast, in TTR-A only expressing flies with the dragged wings, TTR aggregated and did not enter the gel as no soluble TTR-A was detected. Two independent transgenic lines of SAP expressing flies were used in the analysis denoted SAP-18 and SAP-3. (PDF) [file pone.0055766.s003.pdf]
